# A Phase I Clinical Study Comparing the Tolerance, Immunogenicity, and Pharmacokinetics of Proposed Biosimilar BAT1806 and Reference Tocilizumab in Healthy Chinese Men

**DOI:** 10.3389/fphar.2020.609522

**Published:** 2021-01-25

**Authors:** Hong Zhang, Hong Wang, Haijing Wei, Hong Chen, Jingrui Liu, Cuiyun Li, Xiaoxue Zhu, Xiaojiao Li, Jinchen Yu, Yinbo Zhou, Xiaolei Yang, Zhaohe Wang, Min Wu, Yanhua Ding

**Affiliations:** ^1^ Phase I Clinical Research Center, The First Hospital of Jilin University, Jilin, China; ^2^ Jilin Medical Products Administration, Jilin, China; ^3^ Bio-Thera Solutions, Ltd. Guang Zhou, Guangdong, China

**Keywords:** tocilizumab, biosimilar, immunogenicity, pharmacokinetics, intersubject variability tolerance, variability, and pharmacokinetics of tocilizumab biosimilar

## Abstract

**Objective:** The study aimed to explore the bioequivalence of a proposed biosimilar BAT1806 to its reference products marketed in the EU and US (RoActemra-EU and Actemra-US) among healthy Chinese men. The tolerance, immunogenicity, and pharmacokinetics (PK) of the three drugs were also investigated.

**Methods:** In this randomized, double-blind, single-dose, three-arm, parallel study, a single-dose of 4 mg/kg of the reference products, or the biosimilar was administered to the participants. The participants were followed up for 57 days, and PK, immunogenicity, and tolerance evaluations were completed during this period.

**Results:** The PK parameters were similar in all three groups: BAT1806 (*n* = 45), RoActemra-EU (*n* = 42), and Actemra-US (*n* = 42). The 90% confidence intervals (CIs) for the ratios of C_*max*_, AUC_0–*t*_ and AUC_0–∞_ were 86.90–104.41% for BAT1806 vs. RoActemra-EU, 91.70–106.15% for BAT1806 vs Actemra-US, and 90.04–105.53% for Actemra-US vs RoActemra-EU. For all comparisons, the 90% CIs for the C_*max*_, AUC_0–*t*_, and AUC_0–∞_ were within the predefined bioequivalence limit of 80.00–125.00%. The intersubject variability ranged from 14.5% to 21.5%, which was considerably low. Among the participants, 19 (42.2%), 10 (23.8%), and 12 (28.6%) from the BAT1806, RoActemra-EU, and Actemra-US groups were, respectively, found to be positive for anti-drug antibodies, while 14 (31.1%), nine (21.4%), and 12 (28.6%) were positive for neutralizing antibodies. Nevertheless, these antibodies did not affect the drug concentrations, and the outcomes in the bioequivalence tests were similar after sensitivity analysis. Treatment-related and treatment-emergent adverse events (TEAEs) were recorded in 27, 34, and 32 participants in the BAT1806, RoActemra-EU, and Actemra-US groups, respectively. The most common treatment-related adverse events observed were a decrease in neutrophil, and white blood cell counts.

**Conclusion:** The PK characteristics of BAT1806 were similar to those of the reference products, RoActemra-EU and Actemra-US. Both BAT1806 and the reference products exhibited low intersubject variability and similar safety profiles.

**Clinical trial registration number:** http://www.chinadrugtrials.org.cn/index.html, CTR20180039; https://clinicaltrials.gov/NCT03606876

## Introduction

Biological products are diverse, large, complex molecules that typically originate from living cells. Biosimilars are not characterized as traditional small molecules due to their molecular complexity and multifaceted production processes (Agency, 2014; Administration, 2015; Organization, 2015). Although there has been a significant improvement in therapeutic strategies, the worldwide availability of biologic therapies, such as monoclonal antibodies (mAbs), is limited because of the high cost. Biosimilars can improve the overall health outcomes by increasing patient access to biological molecules. Therefore, biosimilars are being developed as alternatives in multiple therapy areas, enhancing patient access, and reducing healthcare-associated costs (Chopra and Lopes, 2017). Biosimilars of existing biologics have been available in the European and United States markets for a decade.

The US Food and Drug Administration (FDA), European Medicines Agency, and the National Medical Products Administration have emphasized a stepwise approach for the development of biosimilars (Agency, 2014). First, biological functions are compared to assess similarities. Next, pharmacokinetic (PK) and pharmacodynamic (PD) characteristics are evaluated. Finally, clinical similarities, including efficacy, safety, and immunogenicity, are assessed using similar approved doses and routes as the reference product (Agency, 2014; Administration, 2015; Organization, 2015).

Tocilizumab is a recombinant humanized IgG1 monoclonal antibody that binds to both soluble interleukin (IL)-6 receptors (sIL-6Rs) and membrane-bound IL-6 receptors (mIL-6Rs) and inhibits IL-6–mediated signaling through these receptors. Several cell types, including T cells, B cells, lymphocytes, monocytes, and fibroblasts, produce IL-6, a pleiotropic proinflammatory cytokine. Synovial and endothelial cells also locally produce IL-6 in inflamed joints, such as in rheumatoid arthritis (Scott, 2017; Biggioggero et al., 2019). Tocilizumab has been globally approved to treat rheumatoid arthritis, giant cell arthritis, polyarticular juvenile idiopathic arthritis, and other conditions. The prevalence of rheumatoid arthritis is about 0.5–1.0% in Western countries and 0.20–0.37% in China. Most patients with rheumatoid arthritis experience a high rate of disability in the first 2–3 years of disease progression. For those who do not receive early treatment, the joint damage can reach 70% within 3 years (Zeng et al., 2008). Therefore, there is an urgent need for corresponding drug treatments such as tocilizumab. Additionally, the availability of biosimilars is the need of the hour to make the drug more affordable to patients at a relatively low price.

Tocilizumab biosimilars are being actively produced globally, as well as in China. The primary structures, post-translational modifications, biochemical characteristics, and biological functions of the tocilizumab biosimilar BAT1806 are similar to those of the reference products (data not published). Preclinical PK and PD studies on monkeys have also demonstrated such similarities (data not published). These studies support the clinical development of BAT1806.

PK studies in humans are crucial for assessing biological analogs and reference products to demonstrate bioequivalence (Administration, 2015). In this study, a single-dose PK study on healthy Chinese men was conducted to assess the bioequivalence between BAT1806 and Actemra [EU-sourced tocilizumab (RoActemra-EU) and US-sourced tocilizumab (Actemra-US)]. Confounding factors such as variability associated with disease conditions, comorbidities, and concomitant therapies are more likely to be absent in healthy individuals. Earlier clinical trials have indicated the therapeutic dose for the reference drugs ranges from 4–8 mg/kg (Socinski et al., 2015; Genentech, 2020). Therefore, a 4 mg/kg dose was used in the current study in line with the sponsor’s earlier clinical trial plans.

Here, the PK profiles of BAT1806 were analyzed and compared with those of RoActemra-EU and Actemra-US. Furthermore, the tolerability, safety, and immunogenicity of BAT1806 were evaluated.

## Methods

### Study Design and Subjects

This study was carried out in the phase I Clinical Research Center of the First Hospital of Jilin University between June 06, 2018, and November 08, 2018 (Chinese Clinical Trial Registry, Registration No. CTR20180039; http://www.chinadrugtrials.org.cn/index.html; ClinicalTrials.gov, Registration No. NCT03606876; https://clinicaltrials.gov). The study protocol was approved by the ethics committee of the hospital. The guidelines of the Declaration of Helsinki and the International Conference on Harmonization Good Clinical Practice Guidelines were followed, and local regulatory requirements were fulfilled. Written informed consent was obtained from all study participants.

This randomized, double-blind, single-dose, three-arm, parallel study was conducted to explore the bioequivalence of the proposed biosimilar, BAT1806 (4 mg/kg), with its reference products marketed in the EU and US (RoActemra-EU and Actemra-US) among healthy Chinese men. The tolerance, immunogenicity, and pharmacokinetics (PK) of the reference products and the biosimilar were investigated.

**FIGURE 1:**
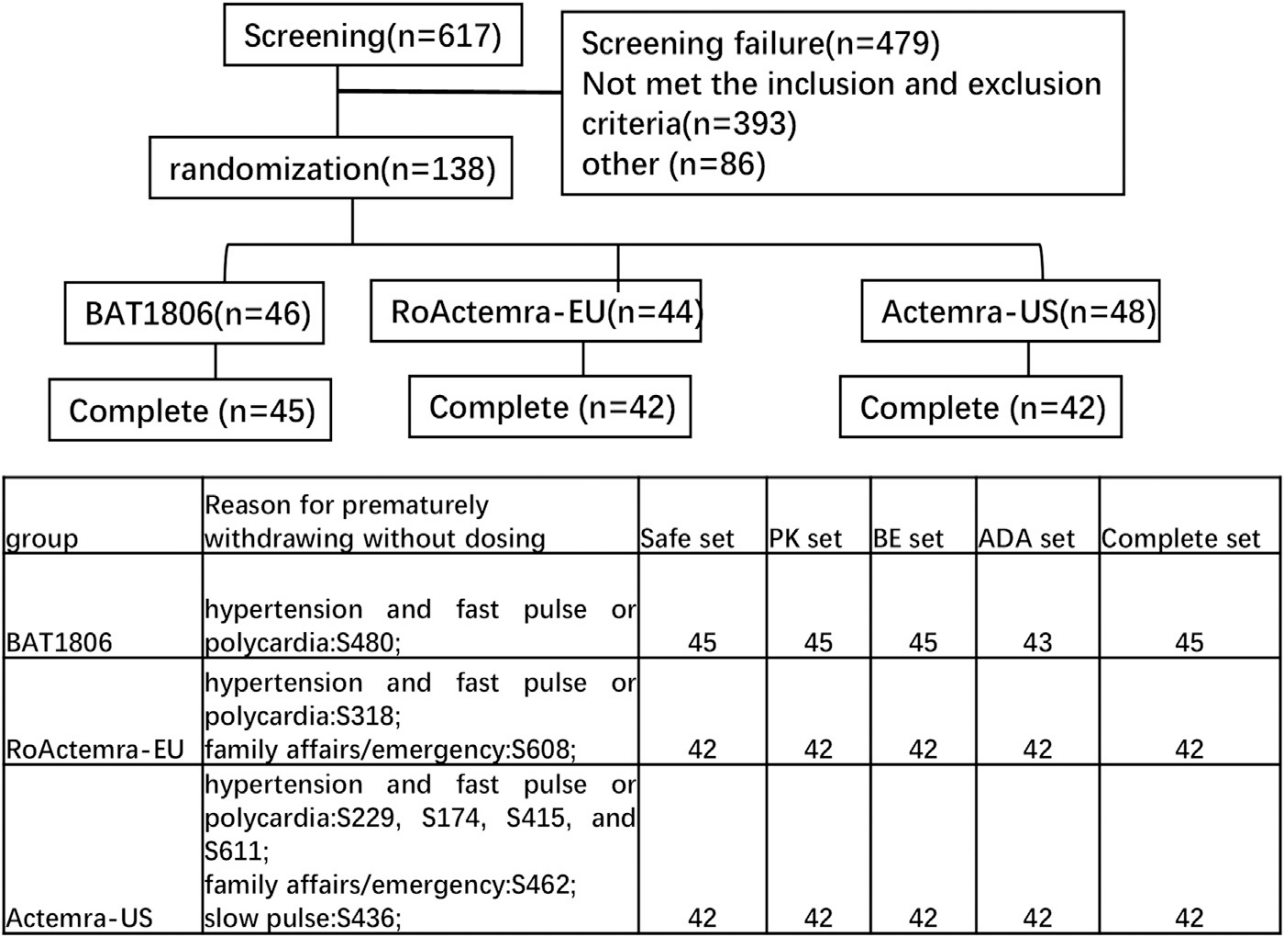
The flow chart of the study and reasons for premature withdrawal before dosing.

Overall, 138 eligible participants were randomly allocated in a 1:1:1 ratio to receive a single intravenous drip of 4 mg/kg BAT1806, RoActemra-EU, or Actemra-US. The subjects were stratified by body weight with a weight interval of 5 kg. Individuals in each of the prespecified weight intervals were equally assigned to the three treatment groups through randomization (Figure 1).

The main inclusion criteria were as follows: 1) healthy men between 18 and 55 years of age; 2) body mass index of 18.0–28.0 kg/m^2^; 3) total body weight between 55 and 85 kg; and 4) normal test outcomes or clinically unremarkable results for blood and urine routine tests as well as hepatic and renal function tests during enrollment; an absolute neutrophil count of ≥1.8 × 10^9^/L; and a platelet count of ≥125 × 10^9^/L. The main exclusion criteria were as follows: 1) Having clinically significant laboratory abnormalities or other clinically indicated diseases (including but not limited to gastrointestinal, renal, liver, neurological, hematological, endocrine, tumor, lung, immune, mental or cardiovascular diseases); 2) patients who participated in any other clinical trials or donated blood within the past 3 months and 3) positive results in T-SPOT^®^ tuberculosis (TB) interferon-γ-release assay, contact with a patient with TB, and/or presenting with suspected symptoms or signs of TB within the past 3 months.

The day before dosing, participants were admitted to the study center. Each participant was expected to stay at the study center for at least 96 h (until day 5) after the initial dose of the investigational product (IP) for safety evaluation. All participants were followed up on days 8, 15, 29, 43, and 57 after infusion for PK, immunogenicity, and safety evaluations. Sentinel staggered dosing was adopted in this study. The participants were initially administered the IP in staggered cohorts, with the first, second, and third cohorts comprising three, six, and nine participants, respectively. After the IP administration, each participant was observed closely for 168 h to assess the drug's safety and tolerability. After the first three cohorts, the number of participants dosed subsequently was not limited.

All participants received a single intravenous infusion of the IP at the same dose (4 mg/kg) for 60 min (±6 min): BAT1806 (Bio-Thera Solutions, Ltd.; Batch number: A0520180402); RoActemra-EU [Roche (United States) and Chugai Pharmaceutical Company (Japan); Batch number: B2065H01]; or Actemra-US [Roche(United States) and Chugai Pharmaceutical Company (Japan); Batch number: B3016B03].

The screening was performed 7 days before the drug dosing date. The screened participants were admitted to the clinical research unit a day before the biosimilars were administered. The participants fasted for at least 8 h before the biosimilars were administered and were randomized into either the test drug (BAT1806) or the reference drug (i.e., RoActemra-EU or Actemra-US) group.

#### PK Evaluations

Blood samples for PK evaluation were collected at 1 h before the initiation of dosing (predose), at 30 min after the start of IP infusion, at the end of infusion (immediately after 60 min of infusion), at 2, 3, 4, 5, 9, and 13 h after the start of the infusion, and at 24 h (day 2), 48 h (day 3), 72 h (day 4), 96 h (day 5), 168 h (day 8), 240 h (day 11), 336 h (day 15), 504 h (day 22), 672 h (day 29), 1,008 h (day 43), and 1,344 h (day 57) after the start of infusion. Serum tocilizumab levels were determined using enzyme-linked immunosorbent assay (ELISA) at the Covance Pharmaceutical R and D (Shanghai) Co., Ltd. (Shanghai, China) (Supplementary Material). A total of 2,581 samples were tested in this study, and a total of 544 samples were below the limit of quantitation (BLQ), which were set to zero, including 130 samples on day 0 and 414 samples at other time points.

PK parameters were determined using a non-compartmental analysis model. The concentration-time data included time to peak (T_max_), the maximum observable serum concentration (C_max_), clearance (CL), half-life (*t*_1/2_), volume of distribution (Vz), and area under the curve (AUC) from zero to the final quantifiable concentration (AUC_0–*t*_) and to infinity (AUC_0–∞_). Actual sampling times were used for PK analyzes. An internally validated software system, Phoenix WinNonLin® v8.0 (Pharsight Corporation, Certara, L.P., Princeton, New Jersey, USA), was used to determine the PK parameters.

#### Immunogenicity Evaluations

Blood samples collected at 1 h before and on 15, 43, and 57 days after drug administration were analyzed for the presence of anti-drug antibodies (ADAs) using an electrochemiluminescence immunoassay (ECLIA). ADA-positive samples were further examined for the presence of neutralizing antibodies (NAbs).

#### Tolerance Evaluations

Hematology, biochemistry, and urinalysis tests were performed, and markers of myocardial damage were examined at screening and on days 2, 5, 8, 15, 29, 43, and 57. Dynamic electrocardiogram monitoring was performed before infusion and at 2 h after infusion. Electrocardiograph (ECG) screening was performed at 60 min predose, and ECG was performed at 5 and 13 h and on days 2, 5, 8, 15, 29, 43, and 57 after infusion. Several evaluations, including physical examination, vital sign verification, electrocardiogram, and common laboratory investigations such as urinalysis and chemistry, were performed to monitor adverse events (AEs). The AEs were recorded and graded according to the National Cancer Institute Common Terminology Criteria for AEs (CTCAE; V.4.03). Participants exhibiting AEs were observed till they reached normalcy or acceptable stability (evaluated both by the principal investigator and sponsor) or were lost to follow-up.

### Estimation of the Sample Size

To ensure an adequate sample size in each group, the sample size was estimated. The following four parameters were considered: ratio (geometric mean of C_max_ and AUC for the test drug vs. the reference drug), power (1−β), significance level (two-sided α = 5%), and intersubject variability (inter-CV) of the C_max_ and AUC. A ratio of 95% and a 90% confidence interval (CI) of 80–125% were set as the bioequivalence limits for the ratio of the geometric mean values of C_max_, AUC0–t, and AUC0–∞ for the test drug vs. the reference drug.

According to recent FDA guidelines, the ratio of the geometric mean (GMR) of C_max_ and AUC for the test drug against the reference drug was set at 95% to obtain 92.8% power (1−β) at the significance level (two-sided α = 5%). The inter-CV was represented by the coefficient of variation (CV). The NQuery 8.3.0.0 (Boston, USA) software was used to determine the sample size (initial: 123; inter-CV of C_max_ for tocilizumab: 25%) (Genentech, 2020). The final sample size was 138 (46 participants per group), allowing for a 10% drop-out rate.

### Statistical Analysis

If the 90% CIs were found to be within 80–125% for C_max_, AUC_0–*t*_, and AUC_0_–_∞_, bioequivalence was inferred. A PK analysis set was used to perform PK analysis in the study population. The analysis of variance (ANOVA) model also included weight as a fixed effect to adjust for its effect on bioequivalence. The safety analysis set included participants who were administered the study drug. Descriptive statistics for the PK parameters and demographic data were estimated. The chi-square test for categorical variables, *t*-test for normally distributed data, and Wilcoxon rank test for data with unknown distribution were used for data analysis. All statistical analyzes were performed with SAS 9.4 (SAS Institute Inc., Cary, NC, USA). A *p-*value of <0.05 was considered statistically significant.

## Results

### Participants

The assigned drugs were administered to 129 of the 138 participants enrolled and included in the safety analysis (Figure 1). Two additional participants were included in the Actemra-US group, whereas two participants were removed from the RoActemra-EU group due to weight stratification. The BAT1806 group comprised a total of 46 participants.

In the BAT1806 group, two participants who were ADA-positive predose were included. One participant from BAT1806 group, two participants from RoActemra-EU group, and six participants from Actemra-US group were withdrawn from the study before the drug was administered for multiple reasons such as family affairs/emergency or hypertension and fast pulse or polycardia, which were not considered to be related to the study drug. (Figure 1). The final per-protocol analysis population included in the safety, PK, BE, and immunogenicity (ADA) analysis set comprised of 129, 129, 129, and 127 participants, respectively (Figure 1). The demographic and baseline characteristics between the per-protocol population and various treatment groups were similar, *p* > 0.05 (Table 1).

**TABLE 1:** Demographics and baseline characteristics.

	BAT1806 (*n* = 45)	RoActemra-EU (*n* = 42)	Actemra-US (*n* = 42)	*p*
Ethnicity (Han, n [%])	45 (100.0)	37 (88.1)	39 (92.9)	0.06
Age (year), mean (SD)	37.4 ± 8.6	35.1 ± 8.4	36 ± 8.3	0.44
Weight (kg), mean (SD)	67.04 ± 8.03	67.57 ± 8.29	66.73 ± 7.48	0.88
BMI, mean (SD), kg/m^2^	23.03 ± 2.3	23.71 ± 2.45	23.39 ± 2.35	0.40

### PK Evaluations

**FIGURE 2:**
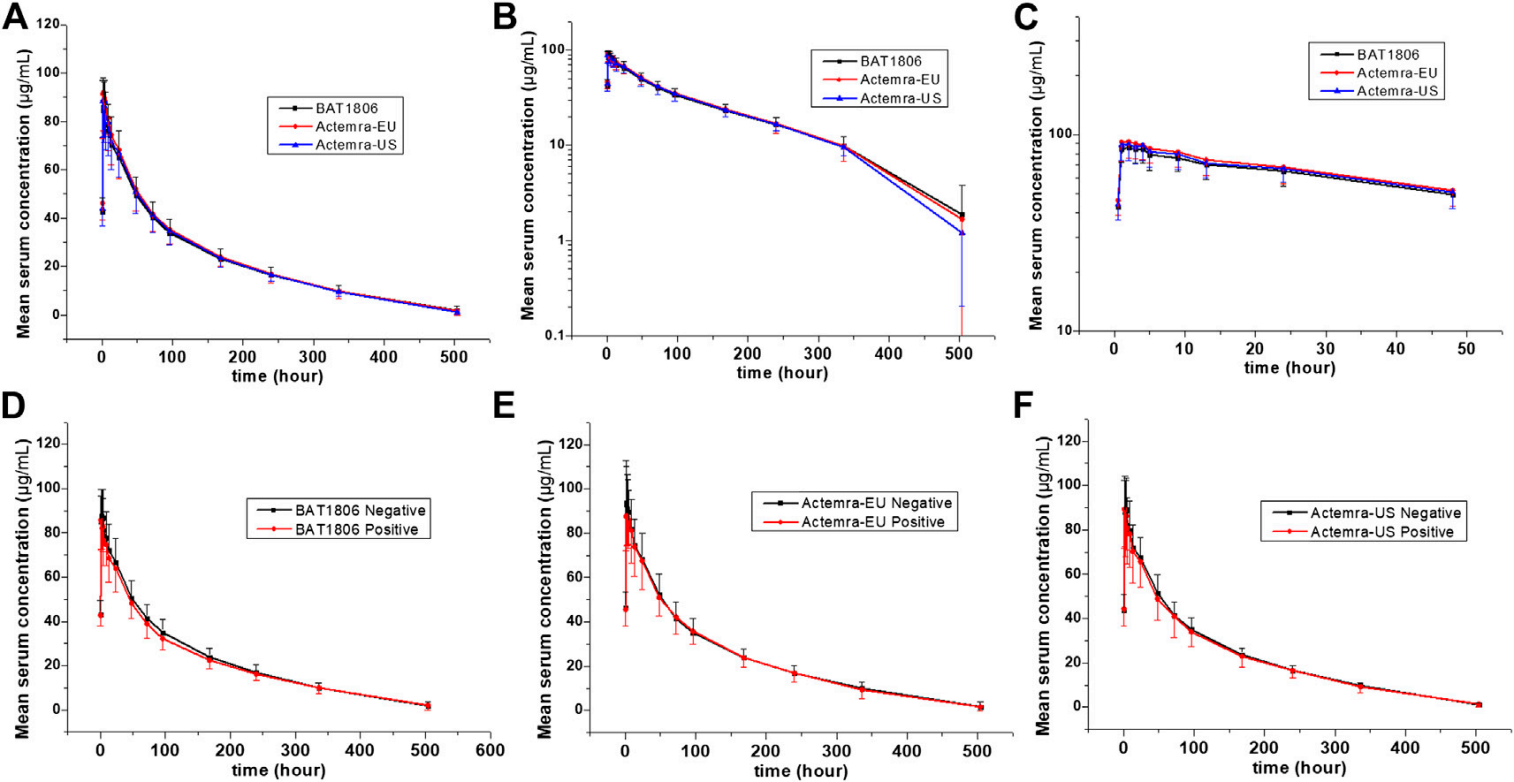
Serum drug concentration-time profiles for tocilizumab and its biosimilar. Mean ± standard deviation values **(A)**; log10 mean ± standard deviation values **(B)**; log10 mean ± standard deviation values within 0–48 h after dosing **(C)**; Mean ± standard deviation values of ADA-positive and negative individuals from the BAT1806 **(D)**; RoActemra-EU **(E)**; and Actemra-US **(F)** groups.

The mean serum concentration-time curves for tocilizumab and its biosimilar demonstrated a multiphase descent; that is, a rapid decline as soon as the infusion ended, followed by a slow elimination phase and a slightly quicker elimination phase at lower concentrations (Figure 2).

The non-compartmental analysis model exhibited slow clearance, longer *t*_1/2_, and small Vz values for tocilizumab and its biosimilars. The median T_max_ values were equivalent across all three groups at 2 h after the intravenous infusion. The estimated tocilizumab geometric mean *t*_1/2_ values were comparable across all treatments, ranging from 72.57 to 89.81 h. The total clearance rate (CL) and Vz values across the three groups were also comparable. The extrapolated AUC values were similar, with <10% variation among the three groups. The mean concentration-time profiles, mean C_max_, AUC_0–*t*_, and AUC_0–∞_ estimates, as well as the inter-CVs, were equivalent, with very low CV values (range: 14.5%–21.5%) (Table 2; Figure 2).

**TABLE 2:** Pharmacokinetic parameters of tocilizumab in each group (GeoMean [CV%] or median [min, max]).

Parameter	BAT1806 (*n* = 45)	RoActemra-EU (*n* = 42)	Actemra-US (*n* = 42)	***p***	**GMR (90%CI)** ^a^	**GMR (90%CI)** ^b^	**GMR (90%CI)** ^c^	Inter-CV (%)	Re-estimate
AUC_0_-∞ (µg*h/mL)	10,840 (16.6)	11,080 (19.5)	10,690 (15.4)	0.73	98.06 (92.10–104.41)	100.82 (95.76–106.15)	97.26 (91.75–103.10)	15.4–19.5	87
AUC_0-t_ (µg*h/mL)	10,260 (17.8)	10,580 (21.5)	10,390 (16.4)	0.78	97.39 (91.01–104.20)	98.18 (92.89–103.78)	99.19 (93.22–105.53)	16.4–21.5	105
C_max_ (µg/ml)	88.28 (14.5)	96.28 (17.6)	91.29 (16.4)	0.03	91.71 (86.90–96.79)	96.25 (91.70–101.03)	95.28 (90.04–100.82)	14.5–17.6	72
T_max_ (h)	2.0 (1.0–9.0)	3.0 (1.0–5.0)	4 (0.98–9.02)	NA					
t_1/2_ (h)	89.81 (32.4)	82.08 (33.5)	72.57 (30.7)	NA					
CL (L/h)	0.02457 (16.4)	0.02421 (18.8)	0.02482 (13.0)	NA					
Vz (L)	3.184 (32.9)	2.867 (32.6)	2.599 (32.4)	NA					
AUCextrapolation (%)	5.35(18.2)	4.51(35.4)	2.80(30.0)	NA					

* Median [min, max].

^a^ BAT1806/RoActemra-EU.

^b^ BAT1806/Actemra-US.

^c^ Actemra-US/RoActemra-EU.

AUC extrapolation, Percentage of AUC0-∞ obtained by extrapolation; not applicable, NA.

The PK parameters were comparable between the groups; the 90% CIs of the ratios for C_max_, AUC_0–*t*_, and AUC_0–∞_ were 86.90–104.41% for BAT1806 vs. RoActemra-EU, 91.70–106.15% for BAT1806 vs. Actemra-US, and 90.04–105.53% for Actemra-US vs. RoActemra-EU, demonstrating biosimilarity. For all comparisons, the 90% CIs of the C_max_, AUC_0–*t*_, and AUC_0–∞_ were within the predefined bioequivalence limit, ranging from 80.00% to 125.00%. The ratio of geometric mean (GMR) 90%CI is a range calculated by ANOVA model parameters, which had no *p* values. The analysis of ANOVA models have *p* values, but they have no relationship with the GMR90%CI. For example, the *p*-value of the ANOVA model of C_max_ between the three groups is 0.03, which means a significant difference (*p* < 0.05). The difference occurred between the BAT1806 and RoActemra-EU group, due to C_max_ was slightly lower at BAT1806 group than that at RoActemra-EU group. However, the difference was acceptable according to the biosimilar guidance of the Food and Drug Administration (Agency, 2014; Administration, 2015; Organization, 2015). The 90% CIs of the C_max_ ratios were 86.90–96.79% for BAT1806 vs. Roactemra-EU, which was within the predefined bioequivalence range of 80.00–125.00% (Table 2).

A larger inter-CV indicated a broader 90% CI. The sample size was reestimated based on the bioequivalence analysis results (GMR and inter-CV), and the reestimated value was less than the enrollment size (Table 2).

### Immunogenicity Evaluations

Two participants from the BAT1806 group were ADA-positive before the administration of the drug. In total, 19 (42.2%), 10 (23.8%), and 12 (28.6%) participants from the BAT1806, RoActemra-EU, and Actemra-US groups tested positive for ADA, respectively, whereas 14 (31.1%), nine (21.4%), and 12 (28.6%) participants tested positive for NAb. The ADA and NAb-positive rates increased over time, especially by days 43 and 57. Nevertheless, the drug concentration was less than the lower limit of quantitation (LLOQ) during those periods. ADA- and NAb-positive rates were similar among the groups 15 days after drug administration (6.7–7.1%). However, at 43 and 57 days after drug administration, the positive rates in the BAT1806 group were higher than those in the RoActemra-EU and Actemra-US groups (Table 3). There was no difference in the ADA-positive rates and Nab among the BAT1806, Actemra-EU, and Actemra-US group (*p* > 0.05) (Table 3).

**TABLE 3:** Summary of immunogenicity assessment (number [%] of subjects with positive antibodies).

Parameter	Time (day)	BAT1806 (n = 45)	Actemra-EU (n = 42)	Actemra-US (n = 42)	Overall (n = 129)	P*
ADA	0	2 (4.4)	0 (0.0)	0 (0.0)	2 (1.6)	0.15
	15	3 (6.7)	3 (7.1)	3 (7.1)	9 (7.0)	0.99
	43	11 (24.4)	9 (21.4)	9 (21.4)	29 (22.5)	0.92
	57	19 (42.2)	10 (23.8)	12 (28.6)	41 (31.8)	0.15
NAb	0	1 (2.2)	0 (0.0)	0 (0.0)	1 (0.8)	0.39
	15	3 (6.7)	3 (7.1)	3 (7.1)	9 (7.0)	0.99
	43	10 (22.2)	6 (14.3)	8 (19.0)	24 (18.6)	0.63
	57	14 (31.1)	9 (21.4)	12 (28.6)	35 (27.1)	0.57

ADA, anti-drug antibody; NAb, neutralizing antibody; p*, The positive rates of ADA and NAb were compared among the three groups.

The serum concentration-time curves for BAT1806, RoActemra-EU, and Actemra-US between ADA-positive and -negative participants were similar (Figure 2). The outcomes for ADA-related sensitivity and the potential influence of ADA on PK analyzes were in agreement with the outcomes from the aforementioned bioequivalence analysis set, resulting in a similar conclusion regarding the PK. Specifically, the 90% CIs for all C_max_, AUC_0–*t*,_ and AUC_0–∞_ comparisons were within the predefined range of 80.00–125.00% for the bioequivalence limit after the exclusion of two participants with positive ADA results before the dosing. This was also the case in all three groups for the same comparisons between participants with ADA-positive results (after dosing) and those with ADA-negative results (Supplementary Tables S1–S3). To summarize, the effect of the immunogenic responses to tocilizumab PK could not be confirmed in this study. The outcomes above demonstrated that ADA did not affect the PK of tocilizumab and its biosimilars.

### Safety Evaluations

Serious AEs (SAEs), deaths, or discontinuations due to AEs were not observed. Overall, 93 (72.1%) participants experienced treatment-related TEAEs, with 27 (60.0%), 34 (81.0%), and 32 (76.2%) individuals from the BAT1806, RoActemra-EU, and Actemra-US groups, respectively (Table 4). The overall incidence rates of treatment-related TEAEs were similar among the three groups. The treatment-related Grade III TEAEs recorded were lowered neutrophil count [*n* = 11 (8.5%)], decreased white blood cell count [*n* = 6 (4.7%)], increased alanine transaminase (ALT) [*n* = 1 (0.8%)] and AST [*n* = 1 (0.8%)], and hypertriglyceridemia [*n* = 1 (0.8%)]. The treatment-related Grade IV TEAEs were reduced neutrophil count [*n* = 2 [1.6%]), hyperuricemia [*n* = 3 (2.3%)], and hypertriglyceridemia [*n* = 1 (0.8%)]. The most common treatment-related TEAEs in all three treatment groups were decreased neutrophil count [*n* = 57 (44.2%)] and decreased white blood cell count [*n* = 35 (27.1%)]. Most of these TEAEs (about 80%) were CTCAE Grade I or II. Participants who exhibited these TEAEs were found to have recovered by the final visit, with 90% recovery reported within a week’s duration from the TEAE onset. The rates of ‘neutrophil count decreased’ in the BAT1806 group was lower than that of RoActemra-EU and Actemra-US groups (26.7% vs. 47.6 and 59.5%, *p* = 0.007), indicating that BAT1806 had less effect on ‘neutrophil count decreased’ than reference products. The incidence of other treatment-related emergent adverse events was not different among the three groups, *p* > 0.05.

**TABLE 4:** Summary of common treatment-related emergent adverse events.

	**Number (%) of subjects**				
	**BAT1806 (*n* = 45)**	**RoActemra-EU (*n* = 42)**	**Actemra-US (*n* = 42)**	**Overall (N = 129)**	P*
Number of participants with treatment-related TEAEs	27 (60.0)	34 (81.0)	32 (76.2)	93 (72.1)	0.07
Investigations	21 (46.7)	26 (61.9)	27 (64.3)	74 (57.4)	0.19
Neutrophil count decreased	12 (26.7)	20 (47.6)	25 (59.5)	57 (44.2)	0.007
White blood cell count decreased	7 (15.6)	12 (28.6)	16 (38.1)	35 (27.1)	0.06
Alanine aminotransferase increased	6 (13.3)	10 (23.8)	7 (16.7)	23 (17.8)	0.43
Aspartate aminotransferase increased	7 (15.6)	8 (19.0)	3 (7.1)	18 (14.0)	0.26
Blood bilirubin increased	0 (0.0)	5 (11.9)	4 (9.5)	9 (7.0)	0.07
Metabolism and nutrition disorders	11 (24.4)	15 (35.7)	8 (19.0)	34 (26.4)	0.20
Hypertriglyceridemia	9 (20.0)	11 (26.2)	6 (14.3)	26 (20.2)	0.39
Hyperuricemia	3 (6.7)	4 (9.5)	4 (9.5)	11 (8.5)	0.85
Blood and lymphatic system disorders	2 (4.4)	3 (7.1)	0 (0.0)	5 (3.9)	0.23
Cardiac disorders	0 (0.0)	4 (9.5)	1 (2.4)	5 (3.9)	0.06
Infections and infestations	1 (2.2)	3 (7.1)	1 (2.4)	5 (3.9)	0.40

P*, The **Number (%) of Subjects** with common treatment-related emergent adverse events were compared among the three groups.

Overall, three individuals reported using a transient concomitant medication, two from the RoActemra-EU group and one from the Actemra-US group. The drugs used were roxithromycin capsules, acetaminophen tablets, cefixime, and fenofibrate. There was no relationship between ADA development and TEAEs in this study. None of the participants demonstrated clinically significant or serious hypersensitivity, anaphylaxis, or injection-site reaction after IP administration. All the drug-induced AEs were reported to the Institutional Review Board of The First Hospital of Jilin University.

## Discussion

This single-dose phase I study demonstrated the bioequivalence of BAT1806 against RoActemra-EU (and Actemra-US) when administered at a dose of 4 mg/kg as an intravenous infusion. A comparison of the C_max_ and AUC values across all three treatment groups through ANOVA indicated that the 90% CIs of the GMRs for these PK parameters were in the range of 86.90–106.15%, which was within the predefined bioequivalence range of 80–125% for the natural log-transformed data for each comparison. Other PK parameters such as T_max_ and *t*_½_ were also similar across all treatment groups. BAT1806, RoActemra-EU, and Actemra-US demonstrated similar safety and immunogenicity profiles. SAEs were absent, and all treatment-related TEAEs were mild to moderate in severity. Local reactions were also absent. All these factors indicated that the three drugs were well tolerated in this population of healthy volunteers. These findings justify further clinical trials of the biosimilars in the next phase (Agency, 2014; Administration, 2015; Organization, 2015; Abdallah et al., 2017).

Factors that differentiate the PK behaviors of tocilizumab from smaller molecules are limited vascular permeability, neonatal Fc receptor recycling, and more frequent receptor-mediated last-step elimination (Schropp et al., 2019). On average, the C_max_ of tocilizumab decreased by about 60% in the first 96 h. This was followed by a slow elimination between 96 and 336 h, and then a relatively quicker elimination between 336 and 504 h (Figure 2). This characteristic behavior is in accordance with higher last-step elimination when drug concentrations decline to considerably low levels, and receptor binding becomes desaturated.

The PK of tocilizumab is characterized by a three-step elimination process, the first being the distribution phase (Dua et al., 2015). The last-step elimination phase for tocilizumab results in an increase in exposure that exceeds the dose-proportional value. Because of the dependence of the total CL on tocilizumab serum concentrations, the t_1/2_ of tocilizumab is also concentration-dependent and varies based on the serum concentration level. Participants in the BAT1806 group who received a dose of 4 mg/kg (mean weight of the participants: 67 kg, dosage: 268 mg [4 × 67]) had a lower CL, longer t_1/2_ (89.81 vs. 39.9 h), and similar T_max_ vs. tocilizumab 162 mg (Roche Products Limited, Welwyn Garden City, United Kingdom). The exposure ratio (AUC ratio of BAT1806-to-tocilizumab 162 mg: 2.39) was more than the dose ratio (268:162 = 1.63), which had been evaluated in an earlier phase I study in healthy participants (Supplementary Table S4) (Zhang et al., 2013).

The PK analyzes conducted thus far for all patient populations demonstrated no correlation between apparent CL and the presence of ADAs (Liu et al., 2015; Genentech, 2020). No effect of ADAs on drug concentration or bioequivalence results was found in this study (Figure 2; Supplementary Table S3). Population PK analysis revealed that body weight is a significant covariate affecting the PK of tocilizumab. When administered intravenously in body-weight–based doses of mg/kg, individuals weighing ≥100 kg were expected to have mean steady-state exposures higher than the mean values for the patient population. Thus, weight stratification was performed to reduce variations in the parameters, although the body weights of the participants in this study were <100 kg. The inter-CV of tocilizumab (<21.5%) was small, and a smaller sample size of 105 individuals, or 35 participants per arm, can be adopted in future studies (Fettner et al., 2019).

**FIGURE 3:**
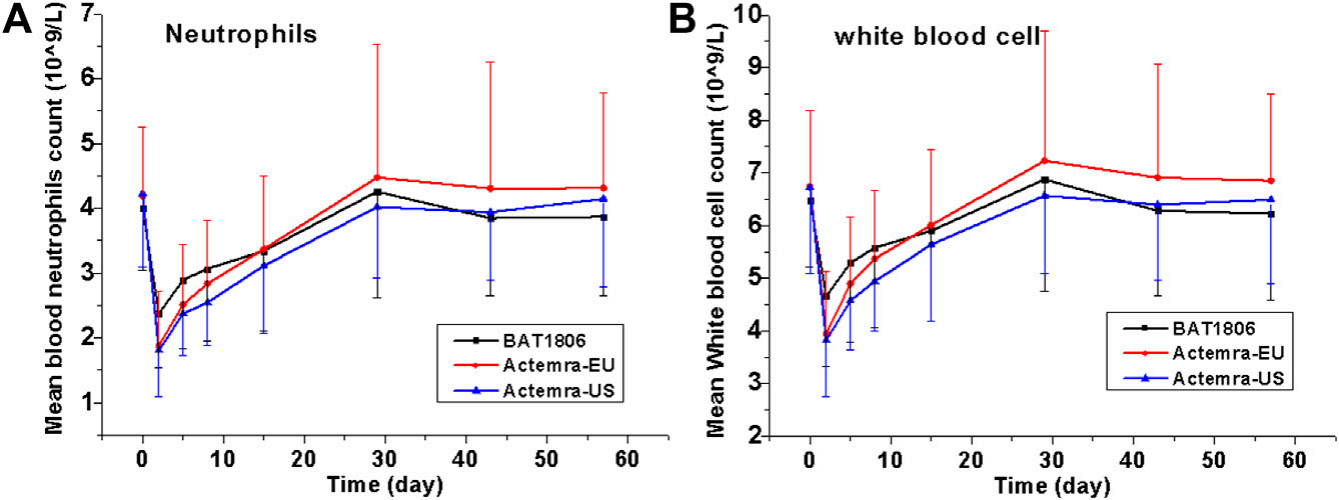
Absolute values for neutrophil and white blood cell counts over time. Data are presented as the means ± standard deviation for the BAT1806, RoActemra-EU, and Actemra-US groups.

The incidence of treatment-related TEAEs in the BAT1806 group (60.0%) was considerably lower than that in the RoActemra-EU (81.0%) and Actemra-US (76.2%) groups. Almost all participants with these TEAEs were found to have recovered by the final visit during the study. The most common AEs (incidence of at least 5%) were reported in the label, such as upper respiratory tract infections, nasopharyngitis, headache, hypertension, increased ALT, and injection-site reactions (Genentech, 2020). Actemra administered in doses from 2 to 28 mg/kg intravenously and 81 to 162 mg subcutaneously in healthy participants was found to reduce the absolute neutrophil counts to the minimum value at 3–5 days after administration. Subsequently, the neutrophils recovered to near-baseline levels in a dose-dependent manner in about 9–17 days (Zhang et al., 2013). Both patients with rheumatoid arthritis and those with giant cell arteritis exhibited a similar pattern in the absolute neutrophil count after Actemra administration (Bastida et al., 2020; Genentech, 2020). As reported earlier, the most common distinct abnormality observed during this study was reduced neutrophil, and white blood cell counts. Neutrophil count decreased on days 2–5, whereas the mean neutrophil count declined to the minimum level on day 2 in these groups. The mean values returned to the baseline levels by days 15–29 in these participants (Figure 3).

Although the incidence of decreased neutrophil and white blood cell counts was high, the CTCAE grades were mostly I or II (about 80%) and the decrease did not predispose the participants to any serious infection during this study. Eleven participants demonstrated Grade III low absolute neutrophil counts on day 2, which improved to Grade II in six participants and Grade I in five participants by day 5. Two participants demonstrated Grade IV low neutrophil counts, which improved to Grade I in one participant by day 5 and in the other participant by day 15. As reported by Nishimoto et al. (Nishimoto et al., 2008), the sIL-6Rs are saturated with tocilizumab as long as the tocilizumab concentration is >1 μg/ml. Thus, IL-6 signaling is completely inhibited, possibly affecting the distribution of blood cells such as neutrophils and white blood cells between the blood and tissues. Thus, these cell counts quickly normalize as the drug concentration drops.

Acute or delayed anaphylactic reactions did not occur in the ADA-positive participants, indicating the absence of product-specific immunogenicity. Although there were no confirmed immunogenic effects of tocilizumab on drug safety and PK in this and earlier studies, we suggest closer examination of the immunogenicity and efficacies of BAT1806 and Actemra in a further phase III study involving a larger population, multiple doses, and a longer study duration because of the availability of increasingly sensitive assay methods for ADA and NAb (Cohen et al., 2018; Gorovits et al., 2018; Zhang et al., 2020). The safety and tolerability of BAT1806 and the reference biosimilars RoActemra-EU and Actemra-US were observed in this study; all AEs were found to be mild to moderate in severity, with no reported SAEs, demonstrating that these products were well tolerated (Boyce et al., 2018).

## Conclusion

In this study, the PK profiles of the tocilizumab biosimilars BAT1806, RoActemra-EU, and Actemra-US were found to be similar. The tocilizumab biosimilars exhibited nearly similar ADA and NAb profiles and also similar safety profiles. The inter-CV of tocilizumab was low among Chinese individuals. These findings support the clinical development of BAT1806 as a tocilizumab biosimilar.

## Funding

This work was supported by the National Major Scientific and Technological Special Project for Significant New Drug Development during the 13th Five-Year Plan Period of China (Project: 2017ZX09304004, 2017ZX09101001–002–004, and 2018ZX09301007005), and Bio-Thera Solutions, Ltd., China.

## Data Availability Statement

The original contributions presented in the study are included in the article/Supplementary Material, further inquiries can be directed to the corresponding authors.

## Ethics Statement

The studies involving human participants were reviewed and approved by The First Hospital of Jilin University, Changchun, Jilin, China. The patients/participants provided their written informed consent to participate in this study.

## Author Contributions

HZ, HW, HC, JY, YZ, XY, ZW, and YD designed the experiment. HZ, MW, XZ, CL, XL, JL, and HW performed the clinic trials. HZ, YZ, and YD analyzed the data. HZ, YZ, and YD wrote and edited the paper and drew the figures.

## Conflict of Interest

JY, YZ, XY and ZW were employed by the Bio-thera Solutions, Ltd.

The remaining authors declare that the research was conducted in the absence of any commercial or financial relationships that could be construed as a potential conflict of interest.
